# Concurrent COVID-19 and *Pneumocystis jirovecii* pneumonia: The importance of radiological diagnostic and HIV testing

**DOI:** 10.1016/j.radcr.2021.09.002

**Published:** 2021-10-02

**Authors:** Amelia Tantri Anggraeni, Soedarsono Soedarsono, Bambang Soeprijanto

**Affiliations:** a Department of Pulmonology and Respiratory Medicine, Faculty of Medicine, Universitas Airlangga, Jl. Mayjen Prof. Dr. Moestopo No. 47 Surabaya, East Java, Surabaya, 60131, Indonesia; b Department of Radiology, Faculty of Medicine, Universitas Airlangga, Surabaya, Indonesia

**Keywords:** COVID-19, SARS-CoV-2, HIV, PJP, Radiological Diagnosis

## Abstract

Coronavirus disease 2019 (COVID-19) caused by severe acute respiratory syndrome coronavirus 2 (SARS-CoV-2) has changed the focus of healthcare and become a public health challenge around the world. The coinfection of SARS-CoV-2 with other microorganisms, including fungi, can cause difficult diagnosis and a worse prognosis. *Pneumocystis jirovecii* pneumonia (PJP) is a common opportunistic infection in human immunodeficiency virus (HIV) patients. However, sometimes the diagnosis is late presented after PJP finding on chest X-ray. We report a 24-year-old man with COVID-19 and PJP. Reverse transcriptase-polymerase chain reaction showed positive for SARS-CoV-2. HIV diagnosis was late presented after PJP finding on chest X-ray examination. HIV serology was positive with an absolute CD4+ count was 16 cells/mm^3^. He was treated with remdesivir IV, methylprednisolone IV, heparin, and cefoperazone-sulbactam IV. He was discharged after being admitted for 25 days. HIV treatment was started in outpatient services. Radiological diagnostic to diagnose concurrent COVID-19 and PJP pneumonia are important, especially in the setting where microscopic examination of sputum or Bronchoalveolar Lavage Fluid (BALF) is not available, or because BAL and sputum induction are aerosol-generating procedures that potentially increase the risk of COVID-19 transmission. HIV testing in COVID-19 patients was also should be considered as part of directed screening in patients presenting with features of PJP, especially for those with unknown HIV status. The determination of an appropriate corticosteroid dose is important to treat both COVID-19 and PJP with severe clinical features. Proper diagnosis and treatment co-infections are urgently needed in this current pandemic to reduce morbidity and mortality.

## Introduction

A current global pandemic of coronavirus disease 2019 (COVID-19) caused by severe acute respiratory syndrome coronavirus 2 (SARS-CoV-2) has spread rapidly causing varying degrees of illness and become a public health challenge all over the world [1], [2], [3]. Comorbidities such as hypertension, diabetes mellitus, obesity, cardiovascular disease, cerebrovascular disease, respiratory disease, kidney disease, and malignancy were reported as risk factors for more severe disease and worse prognosis [4], [5], [6], while many studies reported different results about the seriousness and outcomes of COVID-19 in patients with human immunodeficiency virus (HIV) infection, compared to the general population [3,7,8].

Many drugs specifically targeting SARS-CoV-2 infection are under clinical trial. The coinfection of the SARS-CoV-2 with other microorganisms, such as viruses, bacteria, and fungi, is an important factor in COVID-19, and it can raise the difficulties of diagnosis, treatment, and even increase the disease symptom and mortality [2]. *Pneumocystis jirovecii* pneumonia (PJP) caused by fungal species of *Pneumocystis jirovecii* is a common opportunistic infection in patients infected with human immunodeficiency virus (HIV). However, some people are unaware they are infected with HIV because symptoms might not show for many years, then HIV diagnosis was found at the late stage of infection along with PJP diagnosis [9,10].

We report a 24-year-old man with fever, cough, sore throat, and dyspnea. He was admitted to our hospital which was a secondary referral hospital with reverse transcriptase-polymerase chain reaction (RT-PCR) positive for SARS-CoV-2. Initial chest X-ray showed ground-glass opacity diffuse bilateral suspicion caused by COVID-19 and differential caused by *Pneumocystis jirovecii*. Considering these results, we then decided to perform an HIV test. HIV test showed positive on the next day. Treatment for COVID-19 with coinfection PJP was then adjusted.

## Case presentation

A 24-year-old man was admitted to our hospital as a secondary hospital. This patient presented fever, cough, sore throat, and dyspnea. The vital sign examination revealed a body temperature of 37.1°C, blood pressure of 110/70 mmHg, pulse rate of 96 beats per minute, respiratory rate of 24 breaths per minute, and PaO_2_ 65 mmHg with oxygen saturation of 88% at room air. Oxygen support was directly given and oxygen saturation was 98% with O_2_ simple mask 8 lpm. Laboratory examination on hospitalization day 1 showed a low lymphocyte 13%, high CRP 48.45 mg/L, and procalcitonin 0.28 ng/ml (Table 1). The patient reported no comorbidity.

**Table 1:** Summary of clinical features and laboratory results.

Day of hosp		1	2	3	6	7	10	13
*Vital signs*								
Pulse rate		96	120	114	112	104	92	82
RR		24	26	24	26	24	20	20
SpO2 (%)		98	98	99	94	96	98	97
Note		O_2_ SM 8 lpm	O_2_ NRM 15 lpm					O_2_ SM 6 lpm
*Sign and symptoms*								
Fever				Y				
Temperature (°C)		36.4	37	37.2	36.4	36	36	36.2
Cough		Y	Y	Y	Y	Y	Y	Y
Dyspnea		Y	Y	Y	Y	Y		
Sore throat		Y						
Nausea						Y	Y	
Fatigue					Y	Y	Y	
*Diagnosis*								
RT-PCR		Pos					Neg	
HIV test				R				
*Laboratory results*	Reference range							
CD4 absolute	404-1612				16			
CD4 (%)	33-58				3			
CD8 absolute	220-1129				208			
CD8 (%)	13-39				37.71			
Ratio CD4: CD8	0.69-2.83				0.08			
Hb (g/dL)	13-18	13.9		11.1		11	13.3	14.2
Leucocyte (10^3/uL)	4-10	13.78		13.99		12.96	17.65	15.42
Lymphocyte (%)	25-33	13		13.6		5.1	5.7	9.1
Trombosit	150-450	450		412		528	623	505
CRP	0-3	48.45					8.32	1.14
D dimer (ng/mL)	<0.5	0.54					0.87	0.63
PCT	<0.05	0.28					0.17	
Neutrophil (%)	54-62	80.9		83.9		90.7	88.9	84.2

HIV = human immunodeficiency virus; Pos = positive; R = reactive; RT-PCR = reverse transcriptase-polymerase chain reaction; Y = yes.

**Fig. 1:**
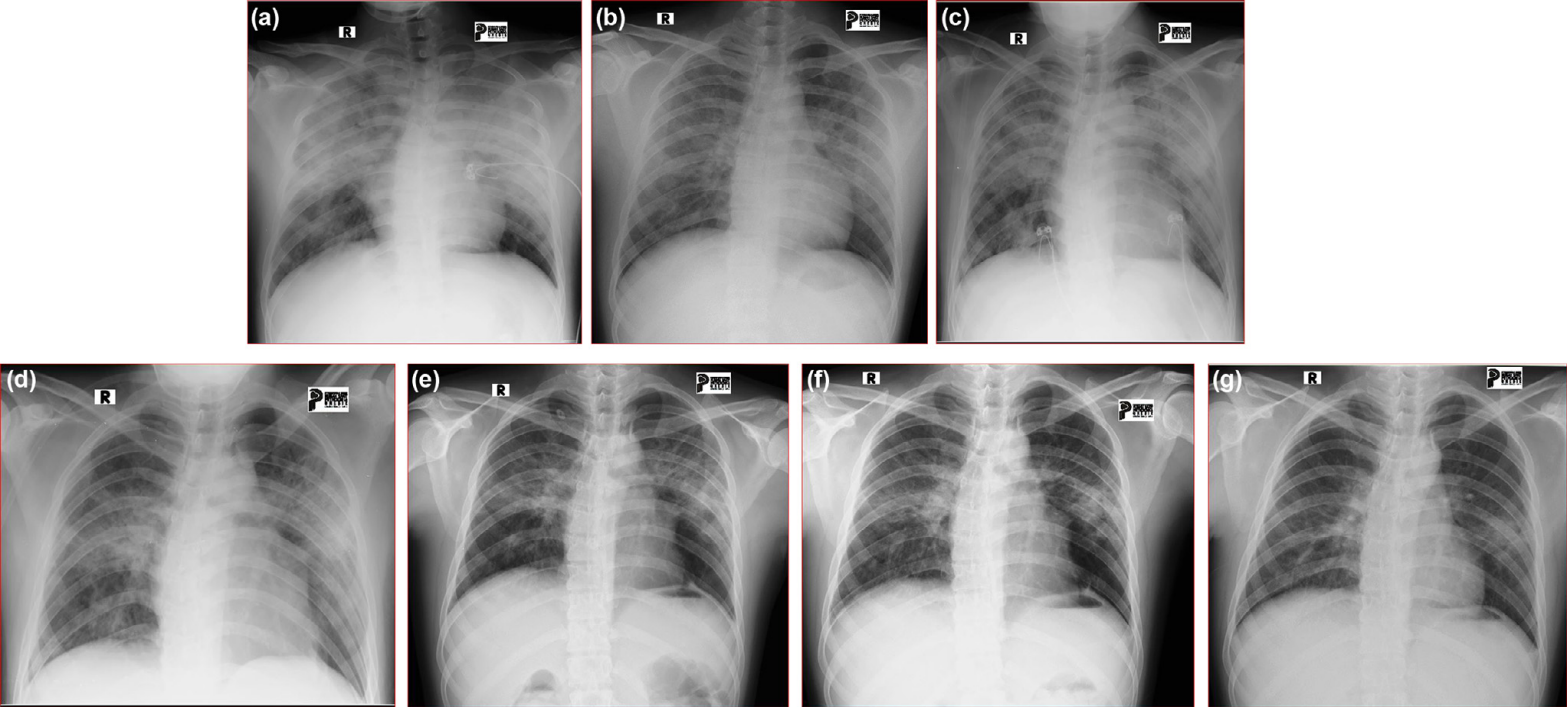
Serial chest X-ray on hospitalization (A. Day 1; B. Day 4; C. Day 7; D. Day 10; E. Day 13; F. Day 18) and discharged on Day 39.

The initial RT-PCR showed positive for SARS-CoV-2 with a CT value of 32.01. The initial chest X-ray examination showed diffused bilateral ground-glass opacity on the upper two-thirds of the lung (Fig. 1A). A diagnosis of COVID-19 and suspicion of PJP was made. This patient was admitted to the isolation room and treated with remdesivir 200 mg QD IV and 100 mg QD IV on the following days, heparin 10000 IU QD syringe pump, vitamin D 5000 IU QD PO, vitamin C 1000 mg QD IV, zinc 50 mg QD PO, methylprednisolone 62.5 mg TID IV (planned for 21 days, tapering down) and oxygen support O_2_ simple mask 8 lpm. Cefoperazone-sulbactam injection was also given due to the presence of bacterial infection signs in the laboratory result.

Considering the opacity pattern on the chest X-ray, an HIV test was performed and showed reactive on hospitalization day 3. PJP diagnosis was confirmed according to the positive results of the HIV test and diffused bilateral ground-glass opacity on chest X-ray. The drug of choice to treat PJP was cotrimoxazole forte 2 tablets PO TID for 21 days. Laboratory tests on hospitalization day 6 showed a low CD4 absolute (16 cells/mm^3^) and CD4 3% (Table 1). This patient had not received HIV therapy at that time. COVID-19 treatment was preferred, it was continued while evaluate and monitor the disease.

Evaluation of serial chest X-ray showed an improvement with the opacity appearance was getting thinner on both lungs. RT-PCR showed negative for SARS-CoV-2 on hospitalization day 10 (Fig. 1). Clinical and vital signs were still fluctuating until hospitalization day 10. This patient was moved to a low care non-isolation room on hospitalization day 12 due to negative results of RT-PCR, SpO_2_ was 97% with O_2_ simple mask 6 lpm. This patient was discharged after being admitted for 25 days. HIV treatment was started in outpatient services. Efavir 600 mg, emtricitabine 200 mg, and tenofovir 300 mg were given. The treatment is still ongoing when this paper was written.

## Discussion

This patient initially presented fever, cough, sore throat, and dyspnea when first admitted to our hospital. COVID-19 diagnosis was confirmed by RT-PCR which showed positive for SARS-CoV-2. Initial chest X-ray of this patient also showed suspected PJP. PJP and COVID-19 have similar characteristics such as fever, fatigue, dry cough, and dyspnea [11]. Chest radiography is the most commonly used imaging tool in pneumonia [12], while RT-PCR is the current gold standard to detect the infection of SARS-CoV-2 [13,14].

*Pneumocystis* cannot be cultured, and the diagnosis of PJP relies on microscopic examination in respiratory specimens obtained from sputum induction or bronchoscopy. Bronchoscopy with BAL is the gold standard procedure to diagnose PJP [9]. Culturing *Pneumocystis jirovecii* is extremely difficult. Confirmation of the diagnosis requires the identification of organisms in sputum or BALF [10]. HIV serology in this patient was positive with absolute CD4+ count was 16 cells/mm^3^ and chest X-ray showed diffused ground-glass opacity, then PJP diagnosis was confirmed without microscopic examination of sputum or Bronchoalveolar Lavage Fluid (BALF) because this examination was not available in a secondary hospital. This decision was also taken under the consideration of COVID-19 transmission risk because BAL and sputum induction are aerosol-generating procedures [13]. PJP is a common opportunistic infection in patients infected with HIV and occurs primarily among persons unaware that they have HIV infection and is an AIDS-defining illness [10]. PJP typically occurs with CD4 counts of less than 200 cells/mm [12]. HIV diagnosis in this patient is late presented after PJP finding on chest X-ray examination.

Although the diagnosis of PJP is only presumptive without respiratory specimens, this finding showed the importance of radiological diagnosis to diagnose concurrent COVID-19 and PJP pneumonia, especially in the setting where microscopic examination of sputum or BALF is not available, or because BAL and sputum induction are aerosol-generating procedures that potentially increase the risk of COVID-19 transmission. In this patient, the finding of radiological diagnosis which showed COVID-19 and PJP also played a role to make a decision on HIV testing.

Initial chest X-ray in this patient showed diffused bilateral ground-glass opacity (Fig. 1). Multifocal ground-glass opacities are the principal finding in both PJP and SARS-CoV-2 infection, making radiographic differentiation potentially difficult, especially in the immunocompromised host [15]. The radiographic finding of PJP typically demonstrates ground-glass opacities and increased interstitial markings. Other radiographic patterns of PJP include a predilection for upper lobes, a preference for central rather than peripheral zones [16,17]. In this patient, the diffused bilateral ground-glass pattern on the upper two-thirds of both lungs made us suspicious of the presence of PJP. Chest CT scan was not examined according to the policy in our hospital that patients with RT-PCR showed positive for SARS-CoV-2 are not allowed to have a chest CT scan examination, this policy aims to reduce the risk of COVID-19 transmission.

Remdesivir was given to treat COVID-19, cefoperazone-sulbactam was due to a high PCT level, heparin was due to a high D-dimer, and adjunctive methylprednisolone therapy for PJP as a secondary infection in COVID-19 patient with severe clinical features. Treating both COVID-19 and PJP was challenging, especially in HIV-infected patients. The administration of adjunctive corticosteroids for the treatment of PJP in HIV patients may reduce the mortality rate of patients in the early phase of the disease [18]. Clinical management of PJP and COVID-19 is different, particularly high-dose corticosteroid therapy is recommended in severe PJP. However, there is no evidence for high-dose corticosteroid therapy in COVID-19 [19]. The use of corticosteroid treatment might not be associated with a lower mortality rate among hospitalized COVID-19 patients. However, in critically ill patients, it could improve outcomes. The effect of corticosteroid treatment on mortality might be limited to critically ill COVID-19 patients [20]. A previous study reported that corticosteroids may be beneficial in severely ill COVID-19 patients [21]. The World Health Organization (WHO) recommended using systemic corticosteroids rather than no corticosteroids for patients with severe or critical COVID-19-infection and against recommended to use corticosteroids for patients with non-severe COVID-19 infection, while CDC and NIH recommended corticosteroid only for hospitalized patients who required supplemental oxygen [13,22]. A study in the UK regarding the use of 6 mg of dexamethasone once a day showed that the efficacy was high and resulted in lower 28-day mortality among patients receiving supplemental oxygen [23]. In this patient, the clinical manifestations showed severe illness, both because of SARS-CoV-2 and *Pneumocystis jirovecii* infections. Administering corticosteroids is absolutely needed for this patient, both for COVID-19 and PJP.

Based on the protocol for COVID-19 in our hospital, we use 6 mg of IV dexamethasone as the standard severe COVID-19 therapy. But in this case, we decided to give a methylprednisolone high dose, considering the low dose of corticosteroid might be useful for COVID-19 but not for PJP. RT-PCR showed negative for SARS-CoV-2 on hospitalization day 10 in this patient, which was different from the previous study by You et al (2020) that stated methylprednisolone could not improve the prognosis of patients with COVID-19. Another study stated that patients without the use of methylprednisolone were more quickly to obtain negative results (11 days) of their nasopharyngeal swab tests of SARS-CoV-2 nucleic acid after treatment, compared to those receiving methylprednisolone (13.5 days) [24].

Monitored during treatment, liver function, renal function, and blood glucose were normal. This patient was discharged and started HIV therapy in outpatient services. HIV patients who are on treatment with antiretroviral therapy (ART) could have successful viral suppression, resulting in undetectable viral load and not a transmissible disease. People living with HIV (PLHIV) are not immunocompromised if ART is maintained [8].

This case showed that a high suspicion of PJP should be considered according to the clinical and radiological features. The clinician should maintain good clinical sense in making the diagnosis, especially PJP diagnosis in addition to COVID-19. The determination of all treatments is important to improve the outcomes in this HIV patient with concurrent COVID-19 and PJP. Previous studies reported different results about the severity and outcomes of COVID-19 in HIV patients. The clinical course of COVID-19 among HIV patients does not seem to be different than it is in the general population, but areas of concern include high inflammatory states, which could result in complications [25]. The presence of multimorbidity and older age appears to be the important factors for severe morbidity and mortality with COVID-19-HIV co-infection [26]. This case was a young HIV patient with concurrent COVID-19 and PJP. The clinical feature of this patient was severe, but he was successfully survived because the accurate diagnosis, appropriate and early treatment had been given for both COVID-19 and PJP.

HIV testing is important for early treatment because HIV patients with well-controlled diseases are not at risk of poorer COVID-19 disease outcomes than the general population [8]. Early detection of HIV infection is a critical factor in controlling HIV. Timely ART is associated with a better prognosis among HIV-infected individuals and lower rates of disease progression. Individuals who present at an advanced stage of immune suppression are at high risk of clinical events and death. Individuals who present at an advanced stage of immune suppression are at high risk of AIDS-related diseases and death [27]. ART was started given in outpatient services with efavir 600 mg, emtricitabine 200 mg, and tenofovir 300 mg.

## Conclusion

Radiological diagnostic is important to diagnose concurrent COVID-19 and PJP pneumonia, especially in the setting where microscopic examination of sputum or Bronchoalveolar Lavage Fluid (BALF) is not available, or because BAL and sputum induction are aerosol-generating procedures that potentially increase the risk of COVID-19 transmission. HIV testing in COVID-19 patients was also should be considered as part of directed screening in patients presenting with features of PJP, especially for those with unknown HIV status. The determination of an appropriate corticosteroid dose is important to treat both COVID-19 and PJP with severe clinical features. Comprehensive diagnosis and proper treatment, both for primary and secondary infections are important in this current pandemic to reduce morbidity and mortality.

## Ethics approval and consent to participate

We are exempt from ethical approval as it is not required in our hospital for a single case report.
